# Macroscopic tensile plasticity by scalarizating stress distribution in bulk metallic glass

**DOI:** 10.1038/srep21929

**Published:** 2016-02-23

**Authors:** Meng Gao, Jie Dong, Yong Huan, Yong Tian Wang, Wei-Hua Wang

**Affiliations:** 1 Institute of Physics, Chinese Academy of Sciences, Beijing 100190, China; 2 State Key Laboratory of Nonlinear Mechanics, Institute of Mechanics, Chinese Academy of Sciences, Beijing 100190, China; 3 School of Energy, Power and Mechanical Engineering, North China Electric Power University, Beijing 102206, China

## Abstract

The macroscopic tensile plasticity of bulk metallic glasses (BMGs) is highly desirable for various engineering applications. However, upon yielding, plastic deformation of BMGs is highly localized into narrow shear bands and then leads to the “work softening” behaviors and subsequently catastrophic fracture, which is the major obstacle for their structural applications. Here we report that macroscopic tensile plasticity in BMG can be obtained by designing surface pore distribution using laser surface texturing. The surface pore array by design creates a complex stress field compared to the uniaxial tensile stress field of conventional glassy specimens, and the stress field scalarization induces the unusual tensile plasticity. By systematically analyzing fracture behaviors and finite element simulation, we show that the stress field scalarization can resist the main shear band propagation and promote the formation of larger plastic zones near the pores, which undertake the homogeneous tensile plasticity. These results might give enlightenment for understanding the deformation mechanism and for further improvement of the mechanical performance of metallic glasses.

Bulk metallic glasses (BMGs) have been studied extensively as potential structural materials due to their excellent mechanical properties such as ultrahigh-yield strengths, large elastic strain limits, high hardness and an ability to be processed like plastics^1^,^2^. Unfortunately, the catastrophic brittle fracture with nearly zero global plasticity is the major obstacle for their structural applications^3^,^4^. To improve the plastic deformation capability of BMGs, great efforts have been made and various methods have been developed^5^,^6^,^7^,^8^,^9^,^10^,^11^. These successful methods for improving compressive plasticity could be roughly divided: intrinsic approaches mainly include modulating elastic modulus^5^,^6^, introducing microscale structural heterogeneity^7^, and minor alloying^8^; extrinsic methods usually enhance the compressive plasticity by the introduction of second crystalline phases^9^, pores^10^, and surface treatments^11^. These methods stimulate the formation of multiple shear bands (SBs) and resist the fast propagation of main SBs, which greatly enhance the compressive plasticity. However, the tensile plasticity of monolithic BMGs is still near zero except for the nano-scale BMG samples^12^ and the special cases for high strain rate^13^.

At room temperature, plastic deformation of BMGs is highly localized into narrow, about 10 nm wide SBs. As shearing deformation proceeds, the friction heat and the drastically reduction of viscosity within the SB strongly weaken the load capacity of BMGs, leading to subsequently catastrophic fracture^14^. Yet the development of such SBs in BMGs does not necessarily result in catastrophic fracture. For example, when these SBs are spatially confined, global plasticity enabled by the formation of a large number of SBs has been observed^15^,^16^; in compressive and bending deformations, multiple SBs formation without crack formation has been reported^17^,^18^. However, under unconfined loading like uniaxial tension, once an SB penetrates the sample and bear the entire load, the system will lose its stability and catastrophic fracture takes place immediately^19^. Thus, the activation of multiple SBs and stabilization of propagation of SBs have become the main scheme to enhance tensile plasticity. Based on this strategy, progress for improving the tensile plasticity have been reported^20^,^21^,^22^,^23^,^24^,^25^,^26^,^27^,^28^. Introducing a second phase has been proved to be a particularly successful strategy to largely promote the tensile elongation ability of the BMG matrix^20^,^21^,^22^. Similarly, Sarac *et al.* introduced a second phase-pore to realize the controllable tensile ductility^23^,^24^,^25^. Meanwhile, Qu *et al.* recently reported that the introduction of the notches and surface artificial indentations can also improve the tensile plasticity^26^,^27^, in which multiple SBs could be promoted in front of notch and surface indentation. Wang *et al.* found that surface mechanical attrition treatment could induce the intense structural evolution and then lead to the formation of gradient amorphous microstructures, which boosts the multiple shear banding and then obtains the superior tensile ductility^28^. However, whether there exists a method to realize the homogeneous tensile deformation in BMGs rather than the inhomogeneous deformation governed by SBs is seldom investigated.

On the other hand, in the nano-scale, the deformation mechanism endures a transition from inhomogeneous to homogeneous deformation not relying on SBs, which results in tensile ductility and even necking^12^,^29^. From this point of view, monolithic BMGs could be intrinsic malleable and ductile under tension. Meanwhile, when the energy state of BMGs is tuned into the higher energy state of the super-cooled liquid, the large tensile plasticity could be also obtained^30^. Similarly, for oxide glass, it has been found that the nanowires show superplastic elongation larger than 200% under moderate exposure to electron beam^31^. These experimental results indicate that the plastic deformation carrier in BMGs may not solely reply on the SBs but a more microscopic deformation units. Many experimental results imply that BMGs are not completely homogeneous in nanoscale, and there exists a lot of dynamic or property defects of flow units (also termed as liquid-like zones or nanoscale SBs)^32^,^33^,^34^. These dynamic defects show low modulus, low viscosity and high atomic mobility. When the fraction of these dynamic defects increases (such as by rejuvenation treatment), the mechanical properties of BMGs such as the plasticity can be largely improved^35^,^36^. A question is then raised: Could we improve the tensile plasticity of BMGs by making the deformation units directly accommodate the plastic strain rather than the SBs? It is challenging to realize above idea considering the SB formation along the main shear plane. However, recent research on the densification and strain hardening under multiaxial loading^37^ implies that the tensile plasticity may be got by complicating the stress field in BMGs. Meanwhile, the stress, which is equivalent to temperature, plays a similar effect on the viscosity, and the yielding could be considered as a stress-induced glass transition^38^. Thus, it is possible for the viscosity of the whole BMG decreases and then approaches the liquid-like state under certain applied stress mode, which leads to the near-homogeneous deformation in BMGs.

The surface artificial defects such as the notch, the indentation printing and the laser shock peening have been verified to induce the stress concentration, which could be used to induce a complex stress field. However, these methods are not readily controllable and do not allow systematic variation of microstructural features, such as phase spacing and volume fraction. As a highly controllable and precise technique, laser surface texturing treatment (LSTT) has been adopted in welding and surface modification of BMGs as well as in cladding of engineering materials with amorphous coating^39^,^40^. Thus, LSTT could be an efficient tool to induce the surface treatments and then create a complex stress field. In present work, a series of designed LSTT pore arrays with different sizes are introduced into typical Zr-based BMG samples. The LSTT samples with different pore sizes display different tensile fracture behaviors and appreciable tensile plasticity is obtained when the size of pore is about 150 ~ 200 μm. The finite element analysis simulations for different LSTT pore arrays were made to analyze the stress distribution evolution. A strategy of stress distribution scalarization is proposed to enhance the macroscopic tensile plasticity of BMGs.

## Results

### Laser surface texturing treatment (LSTT)

**Figure 1:**
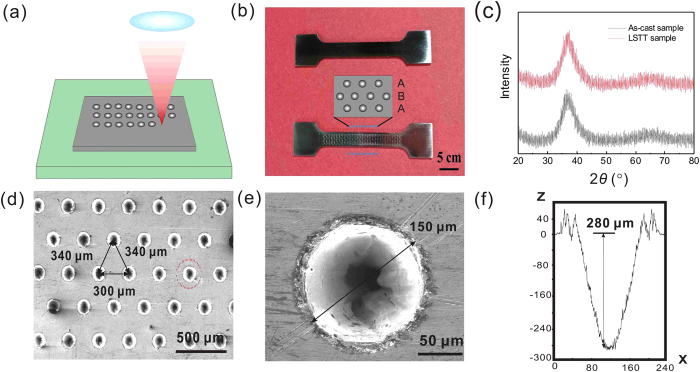
(**a**) The scheme of the experimental set-up of LSTT. (**b**) The optical microscopy images the surfaces of un-LSTT (above) and LSTT (below) samples. In LSTT samples, there are a large number of visible craters and the crater arrangement order is AB-like being shown in the inserted graph. (**c**) DSC curves of the as-cast and LSTT samples. (**d**) The magnified SEM picture in the region of (**b**) circled by the rectangular dashed circle. (**e**) The SEM picture of single LSTT pore. (**f**) The cross-sectional profile picture got by the white light interference profiler.

We designed three kinds of LSTT pore arrays. We showed one of them in the below part of Fig. 1(b), and the as-cast sample for comparison in the above part of Fig. 1(b). Clearly, both the as-cast and LSTT samples are amorphous confirmed by X-ray diffraction in Fig. 1(c). The amorphous nature of LSTT sample can be maintained due to the ultrafast cooling rate of pulse laser during LSTT. Figure 1(d) displays the magnified part of LSTT sample circled by blue dashed rectangular in Fig. 1(b), and the pore arrangement is AB-like pattern [shown in the inserted graph of Fig. 1(b)], which is easier to motivate the formation of multiple SBs^23^. From Fig. 1(e,f), the ratio of the depth and the size of the pores is about 280:150 ~ 1.87 and lies in the range between 1 and 2, which meets our pore profile designing. It is noted that the LSTT samples are different from those of the laser-ablation surface layer in previous research^41^ and the depth of the laser-heating influenced layer is only several hundred nanometers for metals considering the ultrashort laser interaction time (10 fs)^42^. This thin influenced layer does not arise the pronounced effect on the tensile mechanical behaviors compared with the molten layer of the several or hundreds of micrometers during the traditional laser-ablation. What is more, we selectively designed the laser texturing pore pattern on the surface and the shape of the pores were specially designed to the near-cylindrical profile [Fig. 1(f)] to systematically analyze the stress field distribution near the pores by finite element simulation.

### Tensile plastic strain, elastic modulus and fracture strength

**Figure 2:**
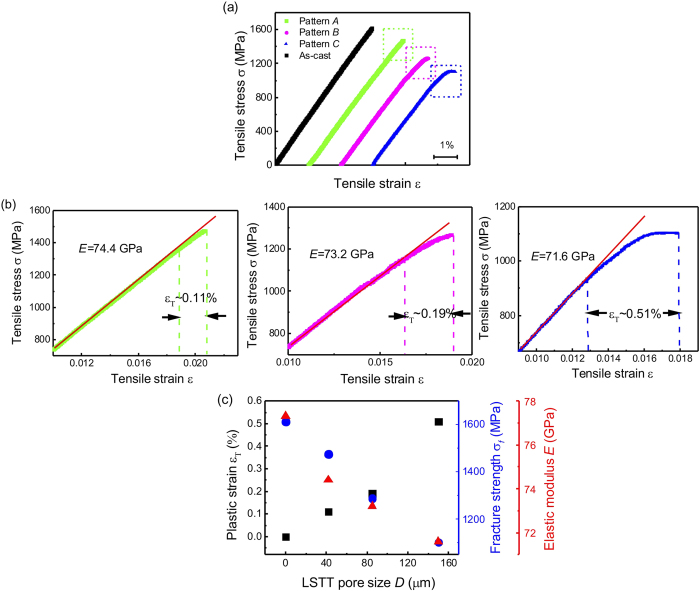
(**a**) Normal tensile stress-strain curves of un-LSTT (black) and LSTT samples (Green, magenta and blue). (**b**) The magnified parts of the tensile stress-strain curves in (**a**) circled by the green, magenta and blue dashed rectangular circles. (**c**) Variation of tensile plastic strain *ε*_*T*_, nominal fracture strength *σ*_*f*_ and elastic modulus *E* with different pore sizes. The value of elastic modulus is measured by fitting the elastic part of the tensile stress-strain curve.

Figure 2(a) shows the typical tensile stress and strain curves of as-cast and LSTT specimens. For the as-cast specimen, no visible macroscopic tensile plasticity and the catastrophic fracture takes place when the tensile strain reaches about 2%; in sharp contrast, obvious tensile elongation appears in the LSTT sample marked with pattern *C* and the pore size of 150 μm. For LSTT samples with the pore size of 42 and 85 μm (pattern A and B), the visible nonlinear tensile stress-strain behavior also appears. The enlarged tensile stress and strain curves corresponding to the parts circled by the green, magenta and blue dashed rectangular circles are also shown in Fig. 2(b). One can clearly see that the nonlinear plastic deformation starts when the tensile strain is ~0.0195 and the tensile plastic strain *ε*_*p*_ is only about 0.11% for the LSTT sample *A*. For LSTT sample *B*, the starting tensile strain of plastic deformation decreases to 0.0164 and the *ε*_*p*_ increases to 0.19%. For LSTT sample *C*, the starting tensile strain of plastic deformation decreases to 0.0128 and the *ε*_*p*_ increases to 0.51%. The above results indicate that *ε*_*T*_ depends strongly on the pore geometry. In addition, no serrated flow in the plastic part of the stress-strain curve of LSST sample *C* in Fig. 2(b), which is the direct hint of SB-governing plastic deformation in BMGs^26^,^27^. The nonlinear plastic part in the stress and strain curves is very analogous to the tensile deformation in the microscale or nanoscale BMGs^12^, which indicates that the homogeneous plastic deformation process may take place within the LSTT BMGs.

With the increase of the LSTT pore size, the tensile plastic strain *ε*_*T*_ increases and the elastic modulus *E*, fracture strength *σ*_*f*_ conversely decreases from Fig. 2(b). The values of *ε*_*T*_, *E* and *σ*_*f*_ with various LSST pore sizes are included in Table.1 and shown in Fig. 2(c). The evolution of *ε*_*T*_ and *E*, *σ*_*f*_ displays the inverse changing trend with the increase of the pore size, which is consistent with previous research^23^. The surface pore array is actually considered as the the second soft phase and the increase of proportion of the surface pores leads to the decrease of *E*. Although *σ*_*f*_ decreases about 30% compared to the as-cast sample, *ε*_*T*_ increases to 0.51% from almost zero of as-cast sample. The above results indicate that to some extent we can tune the tensile plastic deformation ability by designing the LSTT pore stacking.

### Fracture angle and fracture morphology

**Figure 3:**
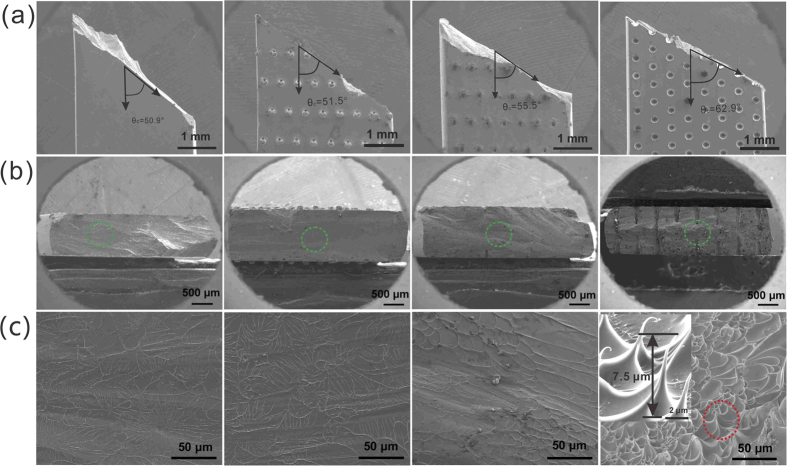
SEM images of the tensile fracture features for as-cast and LSTT samples. (**a**) Macroscopic fracture surface in the lateral surface. The double arrowed lines indicate the fracture angles. (**b**) Macroscopic feature of fracture surfaces in the cross-sectional view. (**c**) A series of high magnitude SEM images showing the typical microscopic fracture morphology corresponding to the parts marked by the green dashed circles in (**b**). The inserted picture gives the magnified graph of the marked part in the last picture of (**c**) and the typical size of the cone-shaped structure.

**Figure 4:**
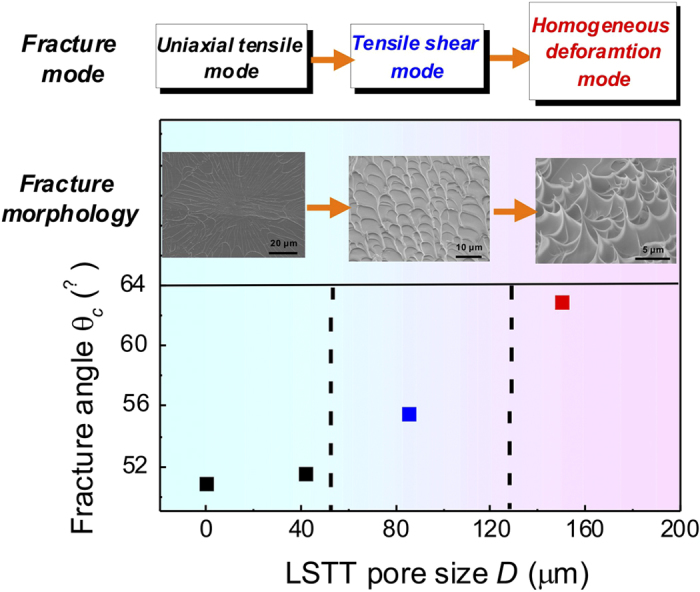
Fracture mode, fracture morphology and the fracture angle variation with the increase of the LSTT pore size.

The LSTT treatment also induces marked change in fracture angle and morphology as shown in Fig. 3. The as-cast sample fails by a single main shear fracture, with a shear fracture angle of ~50.9°, which is consistent with previous research^43^,^44^,^45^. The fracture surface morphology is the typical tensile fracture morphology of firework-like patterns consisting of the core and the radial vein-like pattern in the first pictures of Fig. 3(b,c). This indicates that the normal tensile stress controls the fracture progress. In contrast, the LSTT samples exhibit the larger fracture angles than that that of as-cast samples, and the fracture angle of pattern *A*, *B* and *C* are 51.5°, 55.5°, and 62.9°, respectively [see Table 1], which implies that the LSTT pore array twists the propagation direction of main SBs. Analogous to as-cast sample, the LSTT sample *A* with smaller pore size displays the similar radial-like pattern with smaller size, which indicates the influence of the pore arrays starts to work [second pictures of Fig. 3(b,c)]. For the LSTT sample *B*, the fracture surface displays a vein-like pattern and river-like pattern [third pictures of Fig. 3(b,c)], which is the typical fracture pattern in compression deformation process where the compression and shear stress play the dominant role during fracture. These results suggest the fracture mode has a transition from the uniaxial tensile fracture to compression-like fracture with the change of the pore array and size. For the LSTT sample *C*, the dense micro-scale cone-sharped structures with the size of 7.5 μm appear in the central part between the two opposite surface pores [marked by green dashed circle in the fourth picture of Fig. 3(b)], which only exists in the microscopic BMG samples induced by the size effect such as the micro-scale foils^46^ and the nanoscale samples^29^. These unique cone-sharped structures remind us of the homogeneous tensile fracture morphology in supercooled liquid state of BMGs^47^ and the central part between the two opposite surface pores seems like the liquid state. Previous research^23^,^24^,^37^ have shown that constraints induce the stress concentration to activate the formation of multiple SBs. The SB dominated fracture mode usually express the vein pattern on the main fracture surface^48^. This unexpected unique cone-sharped structures indicates that the fracture mode transition occurs from the usual heterogeneous plastic deformation mode via shear banding to homogeneous deformation in BMGs. The evolution of the fracture angle, the fracture morphology and mode with the LSTT pore size is displayed in Fig. 4 based on the data of Table 1.

### Stress field distribution of LSTT samples with different pore sizes *D*

**Figure 5:**
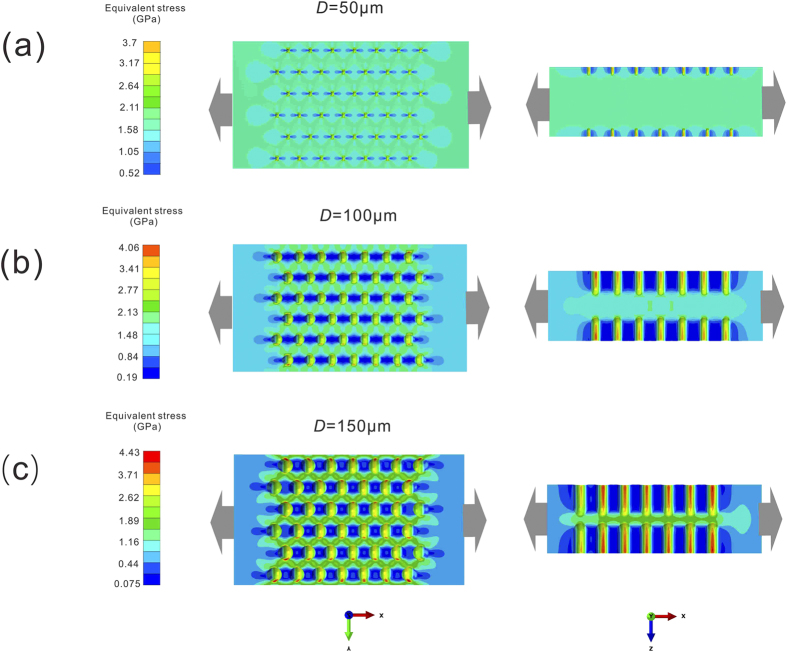
Equivalent stress distribution of LSTT specimens at 2% tensile strain with different pore sizes: (**a**) 50 μm; (**b**) 150 μm; (**c**) 200 μm. The left graphs are the plan view and the right ones gives the cross-sectional view. The gray arrows give the tensile loading direction.

The finite element simulations are adopted to provide explanations for the reduction in fracture strength and appearance of the homogeneous tensile plastic deformation. The numerical results of three LSTT samples (three different pore sizes of 50, 100, and 150 μm) with the elastic strain 2% are displayed in Fig. 5(a–c), in which the elastic modulus of 78.41 GPa and Poisson’s ratio of 0.377 were used for the Zr-based BMGs^8^. Figure 5(a) shows the stress distribution field for LSTT sample with *D *= 50 μm. One can see that most external stress is undertaken by BMG matrix and there appears the stress concentration in the regions near the LSTT pores from both the plan and cross-sectional view. The influence of the LSTT pore is only localized in the regions near pores and the stress field is analogous to that of the as-cast sample. Thus, the fracture features such as the fracture strength, the fracture angle and the fracture morphology do not much change compared to the as-cast ones.

When the *D* increases to 100 μm, the stress field distribution is markedly different in Fig. 5(b). The stress concentrated regions near the pores become bigger, and start to form the grid-like stress concentration zone by hand-in-hand from the plan view. The average stress value near the pores is comparable to the stress value of BMG matrix and the grid-like stress concentration zone starts to carry more the external stress, which indicates that the influence of the LSTT pores has already competed with that of the BMG matrix. In the cross-sectional view, the central parts between the opposite pores undertake the larger stress than BMG matrix and the central parts between the adjacent pores undertake the smaller stress, which produces a compression-like stress field. Thus, this comprehensive stress field disturbs the usual deformation process along the main shear plane and twists fracture angle away from the normal value (~50°). However, this comprehensive stress field does not change the heterogeneous deformation mode via the main SBs in Fig. 5(b) and the main fracture morphology is the vein-like pattern governed by the tensile shear mode.

When the *D* further increases to 150 μm, the regions both near the pores and between the opposite pores firstly reaches yielding compared to BMG matrix and the grid-like stress concentrated regions grow larger. These stress concentrated zones superimpose together and form the yielding zone, in which BMG enters into the liquid-like state and expresses the homogeneous flow behaviors^49^. From Fig. 5(c), one can see that the influenced zones of the LSTT pores has exceed the BMG matrix and the deformation and fracture mode transition happens from the tensile shear fracture to the homogeneous plastic deformation fracture mode. This homogeneous plastic deformation in mesoscopic scale arises the formation of the microscopic cone-sharped structures on the fracture surface of LSTT sample *C* in Fig. 3.

### Stress field evolution of LSTT sample with D = 150 μm in different tensile strains

**Figure 6:**
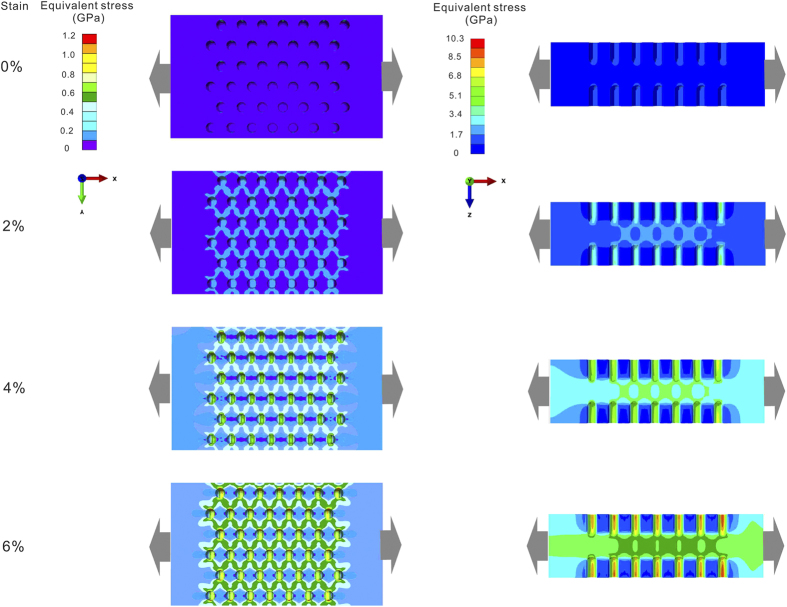
Equivalent stress distribution evolution of LSTT specimen with pore size 150 μm at different tensile elastic strains (from top to down): 0, 2%, 4% and 6%. The left graphs are the plan view and the right ones gives the cross-sectional view. The grey arrows give the tensile loading direction.

We also studied the stress field evolution of the sample with *D *= 150 μm under different tensile strains (0, 2%, 4% and 6%) to understand the evolution of the stress field during tensile deformation in Fig. 6. One can clearly see that the stress concentration should start to take place in the regions near pores and the BMG matrix barely sustains the loading. With the increase of the strain to 2%, the stress-concentrated regions connect each other and form a complex grid-like stress field. The influence of the grid-like stress field plays a dominant role in the following tensile deformation. When the tensile strain reaches 6%, the influenced zones of the grid-like stress field expand to the whole region between the opposite pores from both the plan view and the cross-sectional view. Especially, from the cross-sectional view, the central regions between the opposite pores have entered into the yielding state compared to the BMG matrix. These regions break the main shear plane of the brittle fracture mode without tensile plasticity and lead to the macroscopic tensile plastic deformation in LSTT BMG samples.

## Discussions

Above experimental results and finite element analysis demonstrate that the identical Zr-based BMG specimens with different LSTT pore arrays display quite different tensile fracture behaviors. Under uniaxial tension, applied tensile stress is uniform and it is easier to form a single main SB along the main shear plane, leading to the rapid propagation of SB and the followed brittle failure. For the LSTT samples in this work, the complex stress field (compressive shear stress and tensile shear stress) induced by the LSTT pore array plays a similar role of the second soft crystalline phases^21^,^22^ in activating the production of stress concentrated zones. This complex stress field leads to a complex plastic deformation mechanism in LSTT samples, i.e. the mesoscopic homogeneous plastic deformation near the LSTT pores and the heterogeneous shear banding governed deformation. Thus, the whole stress field is disrupted by the pore array induced complex local stress field, and this effect is equivalent to the transition of a single vectored stress to a multiaxial vectored field, i.e. the stress field scalarization. From this view, stress field scalarization makes the uniaxial tension stress field transform into the multi-axial complex stress field, and then prevents the fast propagation of the main SB and promote the production of the mesoscopic yielding zone, which enhances the tensile ductility for BMGs.

**Figure 7:**
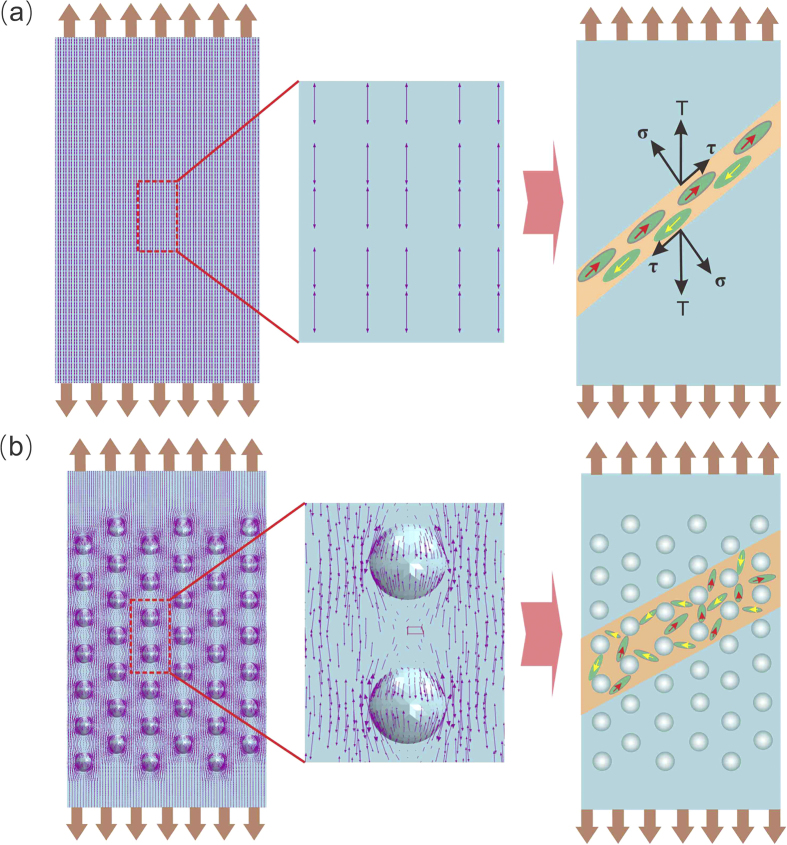
Illustration of the mechanism of the stress field scalarization by LSTT based on the finite element analysis and the flow unit image. (**a**) As-cast sample: (Left) Stress distribution; (Right) Macroscopic shear band formation scheme. (**b**) LSTT sample: (Left) Stress distribution; (Right) Macroscopic shear band formation scheme. The big grey arrows give the tensile loading direction.The modena arrows stand for the projection of tensor stress for elements in the plan view.

Previous works^32^,^33^,^34^,^50^,^51^,^52^,^53^ demonstrated that BMGs is heterogeneous in nano-scale, which consists of flow units and elastic matrix. Upon external loading, the flow units behave like inelastic inclusions and give birth to local plastic events also known as shear transformation zones, which closely correlates with various mechanical behaviors. Based on the flow unit image, the SB can be considered as the assembling consequence of many flow units along the main shear plane. Thus, to clearly understand the physical deformation mechanism of the LSTT BMG samples, a phenomenological picture of stress field scalarization based on the flow units image and the finite element analysis is displayed in Fig. 7. Under uniaxial tensile stress, the stress field displays a near-parallel distribution along the external loading direction for the as-cast sample [left part of Fig. 7(a)]. For this kind of stress field distribution, the total effect of the internal stress field is equivalent to the tensor stress. And it is the tensor stress that directly leads to the formation of a single main SB along the main shear direction, which is prone to induce the catastrophic fracture. In contrast, for LSTT samples, the stress field is twisted in the regions near LSTT pores and the tensor stress with parallel distribution is scalarized [left part of Fig. 7(b)]. The scalarized stress field directly arouses the stress concentration in the regions near LSTT pores, which disrupts the flow units arrangement along the main shear plane. Thus, not only the flow units near the main shear plane are activated, but also the hidden flow units away from the main shear plane can also be excited. Those activated flow units aggregate into the mesoscopic yielding zone near LSTT pores when the *D* reaches the certain value (150 μm in this work). Previous research suggests that the stabilization of SB propagation require that the typical length of the artificial heterogeneous microstructures *D* < *R*_*P*_^25^,^27^,^28^,^43^. *R*_*P*_ is the intrinsic crack tip plastic zone radius, and *R*_*P*_ ~ (1/2π)(*K*_*IC*_/*σ*_*y*_)^2^ (*K*_*IC*_ is fracture toughness and *σ*_*y*_ is the yield strength). For Zr-based BMGs, the value of *R*_*P*_ is about 150 μm. In our case, *D* is the size of LSTT pores. As is shown in Figs 5 and 6, when the *D *< *R*_*P*_ (pore size is about 50 μm), the tensile plasticity is just increased to be ~0.1% and the tensile nominal stress still dominates the fracture process. When *D* is about 100 μm comparable to *R*_*P*_, the deformation mode becomes different and the shear stress starts to play the dominant role. When *D* reaches about 150 μm, the homogeneous plastic deformation near the LSTT pore starts to become obvious, which induces the significantly improvement of the tensile plasticity. This suggests that *D/R*_*P*_ is actually the prominent factor for controlling the stress distribution, and thereby, the fracture strength and tensile plastic strain in BMGs.

We note that the depth of the LSTT pores is an important controlling parameter. The core idea of improving ductility of BMGs by LSTT technique is to tune the stress field distribution for activating more flow units to undertake the external loading. Thus, this work is actually one of a series of methods for stress field controlling engineering in improvement of mechanical properties. The introduced LSTT pore array with the same pore size and arrangement may produce a different stress field distribution when the depth of the pores varies and then leads to a distinct mechanical behavior. Furthermore, the relative thickness of the LSTT pore compared to the thickness of BMG samples may be a key factor when the size of pore is be comparable to the thickness of sample. Therefore, various LSTT patterns could be applied to obtain the corresponding stress field distribution based on the specific BMG sample with wanted mechanical properties

It is worth mentioning that our strategy is significantly different from the previous methods for enhancing the tensile plasticity by promoting multiple SBs^25^,^27^,^28^,^43^. In our case, the carrier of the tensile plasticity is the mesoscopic plastic zone near LSTT pores consisting of flow units rather than multiple SBs. Before the main SB propagates, the regions near the LSTT pores have transformed from the solid-like state to liquid-like state under the compression-shear complex stress field. Although there is only 0.51% tensile plastic strain, the larger macroscopic tensile plasticity might be obtained by further optimizing the profile and spatial distribution of the LSTT pore array, which is our further work. Actually, the methods of introduction of the second crystalline phase, the artificial surface defects and the notches into BMGs^26^,^28^,^29^,^48^ for enhancing the tensile plasticity can also be regarded as other forms of stress field scalarization

## Conclusions

A stress field scalarization strategy is proposed to improve the macroscopic tensile plasticity of BMGs, and the method is proved to be feasible experimentally by designing the laser surface texturing treatments on the surface. The introduced surface pore array can activate the formation of the microscopic plastic zones in the regions near LSTT pores and then connect into a mesoscopic zone when the pore size meets the certain conditions. As a result, the mesoscopic zone undertakes the external stress and then arise the macroscopic tensile plasticity. Under the complex stress field environments, the BMGs display the totally different mechanical behaviors compared to the uniaxial stress field, which provides the in-depth understanding of physical mechanism in different external environmental conditions. Due to the superior forming ability of BMGs within supercooled liquid region, the present strategy can also be readily realized by introduction of various artificial defects on the surface using the superplasticity of the BMG in its supercooled liquid state.

## Methods

### Metallic glasses and the specimen preparation

Zr-based BMG samples with a nominal chemical composition of Zr_64.13_Cu_15.75_Ni_10.12_Al_10_ were prepared by induction melting a mixture of pure metal elements and then casting into Cu mold to form plate shape specimens with dimensions of 1 × 10 × 50 mm^3^. The glassy nature of BMG samples was confirmed by x-ray diffraction (XRD) using a BRUKER D8 ADVANCE diffractometer with Cu K_α_ radiation source and differential scanning calorimetry (DSC) performing under a purified argon atmosphere in a Perkin-Elmer DSC-7. The as-cast BMG plates were polished using 200, 600 and 1200 grit SiC paper successively to remove the thin crystalline surface layer caused by interaction with the mold. The final thickness of polished plates was reduced to about 0.7 mm, with the upper and lower surfaces being parallel.

Dog bone-like specimens for tensile tests with cross section dimensions of 0.7 × 7.0 mm^2^ and a total length of 42 mm were cut from the BMG plates using electric spark line cutting machine and the gauge dimension is 0.7 × 3 × 22 mm^3^. All tensile specimens were polished with 1.5 μm diamond sandpaper to get rid of corrosion pits induced by electric spark line cutting.

### Laser surface texturing treatment

Before tensile tests, the polished dog bone-like specimens were pre-treated by the laser surface texturing treatment technology, LSTT, in the central gauge part and the LSTT set-up sketch is shown in Fig. 1(a). A Picosecond laser TruMicro 5025 was used. The laser produces a beam with a Gaussian energy distribution and operates at 515 nm with a maximum pulse energy of 150 μJ, a pulse duration of 0.01 ns and a frequency of 800 kHz. A scanner head, combined with the laser, allows to reach a high precision during texturing. The BMG specimen was fixed on the movable platform (including the cooling water system with the temperature range between 5 °C and 23 °C). The surface texture can present various forms like streaks, holes and other geometries. In this work, texturing was done in the form of circular pores. After LSTT, the surface micro-pores were then observed by scanning electron microscopy (SEM) conducted in a Philips XL30 instrument and white light interference profiler (BRUKER, Coutour GT). Various laser-induced pore array patterns with different diameters and depths were designed on the tensile specimens. In the practical industrialized applications, the improvement of the mechanical and physical properties by LSTT are largely influenced by the profile (shape, size, density and depth) of pores induced by LSTT^54^. To individually study the LSTT effect on the mechanical properties, we controlled the ratio of the depth and the size of the pores between 2:1 and 3:1 by optimizing the laser parameters and kept the identity of the pores in the spatial arrangement with different sizes.

### Tensile mechanical tests

Uniaxial tensile tests were conducted on the as-cast and LSTT BMG specimens with a constant quasi-static strain rate of about 1 × 10^−4^ s^−1^ under an INSTRON ElectroPuls E10000 All-Electric Test Instrument at room temperature. Strain was precisely and directly measured based on the sample gauge length using non-contacting video extensometer (INSTRON). At least three specimens were measured to ensure that the results were reproducible. The fracture features, such as newly generated tensile fracture surfaces, fracture side surface morphology and fracture angle, were observed by the SEM.

### Finite element simulation

A series of finite element simulations were carried out to probe the mechanical mechanism giving rise to the dramatic tensile ductility enhancement. The dimensions of the model system and pores were designed to be identical to the experiment values to conveniently analyze the difference between the simulation and the experimental results. The number of pores was reduced in tensile direction for saving computing time without changing the final simulation results. Specially, we varied the size of pores to investigate the effect of the heterogeneity induced by the LSTT pores on the tensile mechanical behaviors. Tensile deformation was introduced by applying an X displacement on the right boundary and the left was forbidden to move in X direction, as was shown in Fig. 5. To have an insight into the evolution of the stress field, displacement was imposed by increasing steps 50, 100, and 150 μm, corresponding to the nominal strain 2%, 4%, and 6% respectively.

In the model, the material were treated as isotropic elastic solids, Yong’s modulus and Poisson’s ratio of the BMG were taken to be 78.4 and 0.377, respectively. Previous studies^55^,^56^ have shown that the von Mises criterion is adequate for describing the yield response for amorphous alloys. Therefore, for ease to compare the results among the different types of samples, the von Mises criterion was chosen to be used in the present simulations. The basis set of finite element simulations was chosen to be a four-node linear element. The finite element program, Abaqus (version 6.10, Dassault Syste’mes Simulia Corp., Providence,RI, USA), was employed for the calculation in this work.

## Additional Information

**How to cite this article**: Gao, M. *et al.* Macroscopic tensile plasticity by scalarizating stress distribution in bulk metallic glass. *Sci. Rep.* **6**, 21929; doi: 10.1038/srep21929 (2016).

## Figures and Tables

**Table 1:** Tensile fracture features for as -cast and LSTT samples: LSTT pore size *D*, fracture strength *σ*_*f*_, plastic strain *ε*_*T*_, elastic modulus *E*, fracture angle *θ*_*c*_ and fracture mode.

LSTT pore size *D* (μm)	Fracture strength *σ*_*f*_ (MPa)	Plastic strain *ε*_*T*_ (%)	Elastic modulus *E* (%)	Fracture angle *θ*_*c*_ (°)	Fracture mode
0 (As cast)	1613	0	77.3	50.9	Uniaxial tensile
42	1476	0.11	74.4	51.5	Tensile shear
85	1288	0.19	73.2	55.5	Tensile shear
150	1103	0.51	71.6	62.9	Homogeneous deformation
